# Flipping the hidden curriculum to transform pain education and culture

**DOI:** 10.3389/fpain.2023.1197374

**Published:** 2023-06-19

**Authors:** Aram S. Mardian, Lisa Villarroel, Heidi E. Quist, Lynn E. Chang, Jeffrey S. Mintert, Tiffany N. Su, Amrita Dhanjal-Reddy, Eric R. Hanson

**Affiliations:** ^1^ Chronic Pain Wellness Center, Phoenix Veterans Affairs Health Care System, Phoenix, AZ, United States; ^2^ Department of Family, Community and Preventive Medicine, University of Arizona College of Medicine–Phoenix, Phoenix, AZ, United States; ^3^ Arizona Department of Health Services, Public Health Services, Phoenix, AZ, United States; ^4^ Department of Psychiatry, University of Arizona College of Medicine–Phoenix, Phoenix, AZ, United States

**Keywords:** hidden curriculum, biopsychosocial, biomedical, chronic pain, sociopsychobiological, pain education, patient-centered, culture

## Abstract

Though long-sought, transformation of pain management practice and culture has yet to be realized. We propose both a likely cause—entrenchment in a biomedical model of care that is observed and then replicated by trainees—and a solution: deliberately leveraging the hidden curriculum to instead implement a sociopsychobiological (SPB) model of care. We make use of Implicit Bias Recognition and Management, a tool that helps teams to first recognize and “surface” whatever is implicit and to subsequently intervene to change whatever is found to be lacking. We describe how a practice might use iterations of recognition and intervention to move from a biomedical to a SPB model by providing examples from the Chronic Pain Wellness Center in the Phoenix Veterans Affairs Health Care System. As pain management practitioners and educators collectively leverage the hidden curriculum to provide care in the SPB model, we will not only positively transform our individual practices but also pain management as a whole.

## Introduction

In 2011, the U.S. Institute of Medicine (IOM) called for a cultural transformation of attitudes toward pain, explaining that a person’s pain experience is unique and influenced by a variety of factors beyond the biomedical ones. Prominent recommendations from the IOM report, emphasized by many additional experts, included an integrated approach to pain management with an emphasis on promoting self-management (1, 2). Over a decade later, biomedically focused pain management is still the norm, and this outdated model’s attempt to provide a quick fix with medications or procedures continues to fall short of the IOM’s recommendations. With all the discussion over the intervening years of how to change and improve pain management, why has change been elusive? We argue that the hidden curriculum that perpetuates the biomedical model in pain education and practice is at the root of our collective resistance to true cultural change. We submit that recognizing the undesired aspects of the hidden curriculum and intentionally modifying them will transform pain management culture from its current state to one that is conducive to the patient-centered sociopsychobiological (SPB) model.

## The history of the hidden curriculum

Identified by Hafferty and Franks, “the hidden curriculum” was described as the vehicle by which students of medicine learned “the values, attitudes, beliefs, and related behaviors deemed important within medicine” (3). The concept described “a set of influences that function at the level of organizational structure and culture” that existed outside of the formal curriculum (4). A 2018 scoping review traced this idea of a “hidden curriculum” through over twenty years of discussion in the medical literature, noting that the effects of the hidden curriculum were often more influential than the formal curriculum and sometimes harmful (5). One study, for example, reported that learners exhibited decreasing empathy as they observed their clinical teachers (6). Because of its pervasive yet indirect influence, the hidden curriculum has been a widely discussed and repeatedly studied aspect of medical education at large and as well as in specific medical specialties (5).

Pain management is one of the specialties where the hidden curriculum has been explored, though not extensively. One Canadian study offered insight into the hidden curriculum when medical students reported observing physicians treating patients with pain as a “nuisance” rather than “taking the time to practice good pain management” (7). Another study interviewed 13 medical students about their pain management education and found that the hidden curriculum taught students that patients with chronic pain are “too difficult” (8). Furthermore, a study in the UK interviewed 21 medical students and found that the hidden curriculum modeled dismissive behavior toward patients with fibromyalgia (9). Similarly, a 2017 scoping review suggested that the hidden curriculum portrays patients with chronic pain as “a distinct downside of primary care practice in general” (10).

While discussion of the hidden curriculum was initially mostly focused on identifying the negative aspects of medical culture passed on to trainees in clinical training, recommendations followed for the medical community to take action and reform this negative hidden culture (11). For example, Senior wrote of the intentional hidden curriculum he strives to embody for the benefit of his students (12), and Webster et al. wrote that the hidden curriculum can be seen as a “fertile ground for critical reflection on how socialization processes could be better structured and enacted” (10). Others have suggested that working with the hidden curriculum can even be “exciting” when trainees are encouraged to critically evaluate and explore the hidden curriculum for themselves, with the aim of helping them feel empowered to act differently from those they may have observed (13). Indeed, a modern approach to pain education expects a certain degree of disconnect between the formal curriculum and what learners observe in clinical practice, and it embraces rather than eschews that didactic dissonance (14, 15). As the hidden curriculum has become ever more visible, educators have moved to put it on intentional display for each trainee to individually and critically explore. We would add that the hidden curriculum, and the culture it represents, ought now to be treated critically not only by trainees, but by each member of a pain management team. The hidden “values, attitudes, and beliefs” (3) embodied in the biomedically focused pain management culture would greatly benefit from careful and critical review.

## Today’s hidden (biomedical) curriculum in pain education

The hidden curriculum of pain management education is usefully explored by a critical evaluation of the biomedical model. On rotations, trainees often see preceptors searching for a pathoanatomical cause of a patient’s pain (i.e., a single pain generator) and treating that generator as the primary source of a patient’s pain. This method, whether or not physicians themselves are conscious of it, suggests attitudes and beliefs out of sync with the IOM’s recommendations that healthcare practitioners consider a multiplicity of factors that influence the generation of pain (1). Trainees might also observe physicians’ emphasis on imaging (16) as another manifestation of implicit beliefs about pain generators, despite the poor correlation between anatomic abnormalities and pain (16–20) and the lack of an identifiable source of pain in nearly all patients with low back pain (21). Once a pain generator is identified, students are likely to see a formulaic approach to managing pain, often driven by a patient’s distress level, which includes spending the majority of the appointment time describing and recommending the next most potent medication or the next most invasive treatment. The allocation of time conveys to the patient, and to the trainee, that moving on to more invasive treatments is more important than exploring the social, psychological, and physical factors that are primary drivers of the experience of pain and pain-related disability. Unfortunately, trainees may also observe physicians feeling pressured to “do something” when patients are in high distress and repeatedly reporting lack of improvement with passive treatments. Indeed, physicians are likely to recommend progressively more invasive and more potent treatments, even though research suggests that this escalating approach does not often yield improvement (22, 23).

As trainees observe physicians relying on a pathoanatomic pain generator and responding to patient distress in order to direct a treatment plan, learners internalize that physicians are the active party in pain management—the one who assumes responsibility for both the treatment and subsequent outcomes—while patients are merely passive recipients. This approach contrasts with the emphasis on self-management and collaborative treatment planning promoted by the IOM. If a patient’s pain does not improve after multiple interventions aimed at the pain generator(s), instead of empowering the patient to move forward, a physician might determine that the patient does not, after all, have “real pain” or that the pain is “in their head,” and they might comment to a trainee that “the pain must be supratentorial.” This type of interaction perpetuates the outdated implicit belief that all pain has a physical pain generator that can be fixed and discounts the true driver of the chronic pain experience and pain-related disability that patients can learn to manage: the complex interplay of social, psychological, and biological factors.

Counteracting a practice replete with hidden values, attitudes, and beliefs at odds with current evidence is a daunting task, especially given that this particular hidden curriculum has resisted reform for decades. However, we argue that pain management’s hidden curriculum, as well as the specialty as a whole, can move forward if a more helpful process is consciously adopted.

## Defining the sociopsychobiological model

The sociopsychobiological (SPB) model is the model that addresses the pitfalls of the biomedical model as well as those of past attempts to implement the biopsychosocial (BPS) model. In 2014, Carr and Bradshaw (24) proposed the SPB model and described it as a necessary “flipping” of the BPS model that was first proposed by Engel in 1977 (25). While Engel’s intent was to move beyond the reductionist view that ignored sociological and psychological factors’ impact on the pain experience, Carr and Bradshaw argued that the way the BPS was actually implemented continued to give substantial attention to biological factors while the psychological and social ones were viewed as “messy and disturbing” and mere “distractions” (24). Carr and Bradshaw instead advocated for a top-down, SPB approach where pain is seen as “a population-based social phenomenon” and students are sensitized to “complex everyday pain and pain treatment-related problems such as disability certification, mental health issues, family embroilment, and diversion of analgesic medication” (24). In the SPB model, social and psychological aspects of pain are seen as integral and higher-order components rather than as afterthoughts, and patients are encouraged to share with their healthcare practitioner the responsibility of making and carrying out their treatment plan (26). Because of these qualities, the SPB model, if fully implemented, would reflect the change in culture called for by the IOM years ago. We submit that the hidden curriculum, though currently acting as a hindrance, can instead be harnessed to effect positive cultural change.

## Leveraging the hidden curriculum to transform culture

We propose that the hidden curriculum can be leveraged to transform the culture of pain management, specifically from a biomedical to a SPB approach. The theory behind our proposal is based on Implicit Bias Recognition and Management (IBRM), which is a curricular approach for driving change that was developed by researchers working to target implicit bias—the unconscious and automatic evaluations that impact an individual’s decision-making and behaviors (27). IBRM involves first recognizing that implicit biases and beliefs exist, thus making them visible. Then, after self-reflection and critical appraisal, intentional actions are taken to implement desired behaviors based on the insights gained (28–30).

Application of this strategy to pain management would require first acknowledging that there is a gap between the approach to pain care that is recommended by the IOM (e.g., the SPB model) and common practice (the biomedical model). Next follows a recognition and a “surfacing” step in which undesired implicit values, attitudes, and beliefs (such as focusing on pain generators and imaging, routinely relying on progressively more invasive treatments, and fostering a passive role for patients) are explicitly identified. Finally, intentional efforts are required to implement structural changes based on these insights gained, thus reframing the hidden curriculum to embrace a SPB approach.

Examples of structural changes that can reframe the hidden curriculum are exhibited by the Phoenix Veterans Affairs Health Care System’s Chronic Pain Wellness Center (CPWC). The CPWC’s structure reflects the SPB model in many ways. First, timing and staffing of patient appointments reflect the clinic’s belief in the complexity of chronic pain as opposed to a single “pain generator.” Whole person assessment and treatment is supported by allocating ample time (60–90 min) for initial patient evaluations and creating co-disciplinary appointments. During a co-disciplinary visit, two clinicians from different disciplines meet with a patient simultaneously (e.g., a physician and a pain psychologist) to fully assess all sociopsychobiological factors contributing to the pain experience. Additionally, interdisciplinary pain rehabilitation groups (31, 32) that bring together a physical therapist, pain psychologist, recreation therapist, and dietician are the cornerstone treatment for patients with high-impact chronic pain.

Second, once a patient chooses to engage with the CPWC, they are guided through an active self-management approach to pain management that favors evidence-based active therapies rather than a focus on identification and treatment of pain generators. A collaborative treatment plan is developed with the patient that emphasizes active therapies and aims to empower and equip the patient to meet their functional and quality of life goals. Passive therapies are selected to support active care and are prioritized to favor higher value treatments, with “value” defined as evidence of benefit divided by the product of cost and harm (31). Invasive procedures are reserved for patients with indications supported by clinical practice guidelines (33), and clinicians work with patients to help them gradually reduce their reliance on procedures and other high-risk therapies (e.g., high-dose opioid therapy).

Third, the CPWC structures time and space for team members across disciplines to develop shared attitudes and beliefs about the importance of using the SPB model and interdisciplinary teams to treat chronic pain. The clinic sets aside protected time for integrated treatment planning and reflection during weekly team meetings for case conferences and in a separate weekly Balint group. Balint groups explore the experience of both the patient and the healthcare practitioner(s) from a recent memorable interaction and offer space for team members to engage in self-reflection and develop into a more empathetic and effective clinician. During these sessions, one case is presented and team members focus on building an awareness and understanding of how a clinician’s emotional state might influence an interaction with a patient; team members alternate acting as facilitator. Balint groups have been associated with a number of positive outcomes, including burnout prevention (34–38); increased competence and improved relationships with patients (39); and increased meaning in work, reduced depersonalization and emotional exhaustion (40). The CPWC’s continual focus on interdisciplinary teamwork facilitates a cohesive culture and emphasizes the SPB value that patients receive whole person care from the team rather than from a single healthcare practitioner focused on a single pain generator.

CPWC intentionally uses additional structural characteristics to promote the SBP model: developing shared language, promoting continuous learning, and facilitating team development. Team members are taught patient-centered pain language that promotes a sense of safety, reduces a sense of danger (41, 42), and focuses on patients’ goals for improving function and quality of life. Within the clinic, the team regularly discusses shared values, which include maintaining high levels of mutual respect, addressing challenges together, and developing the intellectual virtues of curiosity, humility, courage, and creativity (26). The team comprises healthcare professionals from the following disciplines: pain psychology, addiction, nursing, physical therapy, recreation therapy, complementary and integrative medicine, dietetics, health coaching, clinical pharmacy, and pain medicine. All team members are encouraged to complete shared reading assignments, including the SPB-focused *Arizona Pain and Addiction Curriculum* (26), as well as content about cognitive behavioral therapy for chronic pain (43, 44), acceptance and commitment therapy (45, 46), and pain neuroscience education (41, 47). Ten to twenty-minute mini didactic sessions in which rotating team members from all disciplines share information about their approach to pain assessment and treatment are regular components of weekly team meetings. Learning more about the unique approaches of each discipline fosters a high degree of mutual respect within the team, which is a foundational value for high functioning interprofessional teams (48). Lastly, an annual team retreat is intentionally used to regularly recalibrate and strengthen our SPB approach. Essential to each of these learning activities is an environment where team members feel engaged, empowered to ask questions, and comfortable offering dissonant opinions (49). Team leaders overtly work to create such a space, and that environment, combined with an iterative process of shared learning, helps the CPWC team continue to recognize new gaps, make visible the values and beliefs behind them, and implement structural changes to address them.

The culture within the CPWC is deliberately shared with trainees. Trainees at the CPWC will hear that patients with pain are “complex” rather than a “nuisance” and that they “may benefit from a higher level of care” rather than that they are “too difficult.” Instead of modeling the biomedical model’s reductionist approach of looking for a simple solution, the CPWC invites trainees to acknowledge the real complexity of chronic pain and to work as part of an interdisciplinary team that partners with the patient to manage it. Intentional structural changes have reformed the hidden curriculum, and the hidden curriculum at the CPWC is now one we hope trainees will take with them and replicate.

## Discussion

Today’s dominant pain management culture, like chronic pain itself, has no easy fix. However, by flipping the hidden curriculum and deliberately creating a SPB-supportive culture and practice structure, as illustrated by examples from the CPWC provided above, we propose that pain management clinicians and educators can take similar steps to initiate change. Deliberately flipping the hidden curriculum will likely start on a smaller scale than our CPWC example. For example, changing the language about pain that is used with patients and trainees may be the most feasible initial step. Subsequent steps might include introducing small structural changes, such as creating didactic sessions, team meetings, or patient visits that include clinicians from multiple disciplines. Eventually, teams may establish group discussions that serve as a forum for intentional dialogue about specific pain management values, attitudes, and beliefs. Determining how best to facilitate change on a large scale is then the next step.

We acknowledge that the healthcare system at large is often working against this cultural change. In a fee-for-service model that prioritizes reimbursement for brief visits and invasive procedures, time spent learning about the social, psychological, and physical complexities of our patients, time for interdisciplinary collaboration, and time to empower and equip our patients with the right tools is not time that is financially rewarded. However, a promising development in reimbursement reform aimed at incentivizing whole person, interdisciplinary care was finalized by the U.S. Center for Medicare and Medicaid Services (CMS) on November 1, 2022, effective January 1, 2023. With the introduction of new codes (G3002 & G3003), CMS aims to “prompt more practitioners to welcome Medicare beneficiaries with chronic pain into their practices, and encourage practitioners already treating Medicare beneficiaries who have chronic pain to spend the time to help them manage their condition within a trusting, supportive, and ongoing care partnership” (50). Armed with a process for leveraging the hidden curriculum for cultural change and the early steps favoring reimbursement reform, we urge pain management clinicians and educators to embrace the curiosity, humility, courage, and creativity required to move forward.

Additional examples of implementing the SBP model of pain management, particularly in a non-Veterans Affairs setting, are needed. Further examples of didactic content for ongoing group pain education and developing shared language would also be helpful. Finally, studies of the values, attitudes, and beliefs about pain exhibited by trainees who learn in practices exhibiting either the biomedical or the SPB model may also guide next steps.

## Conclusion

A cultural transformation of pain management practice and education has continued to elude our best efforts for over a decade, in part because of an entrenched hidden curriculum that perpetuates a biomedical model of managing pain even when a more comprehensive approach is taught in the classroom. By identifying and making visible the hidden values, attitudes and beliefs that perpetuate the current culture, we will be able to take intentional steps to create a new culture, one that will support the SPB model. As more and more clinicians and practices leverage the hidden curriculum as a tool for deliberate change, we can expect progress toward the long-sought transformation of pain management as a whole.

## Data Availability

The original contributions presented in the study are included in the article; further inquiries can be directed to the corresponding author.
